# Lamprey IGF-Binding Protein-3 Has IGF-Dependent and -Independent Actions

**DOI:** 10.3389/fendo.2016.00174

**Published:** 2017-01-18

**Authors:** Yingbin Zhong, Cunming Duan

**Affiliations:** ^1^School of Biology & Basic Medical Sciences, Medical College, Soochow University, Suzhou, China; ^2^Department of Molecular, Cellular, and Developmental Biology, University of Michigan, Ann Arbor, MI, USA

**Keywords:** IGFBP-3/Igfbp-3, sea lamprey, ligand-binding domain, nuclear localization, transactivation activity, BMP2/Bmp2

## Abstract

Insulin-like growth factor-binding proteins (IGFBPs) are multifunctional proteins that possess IGF-dependent and -independent actions. Recent studies suggest that its IGF-independent action appeared early and that the IGF-binding function may have been acquired later in evolution. The timing of the emergence of IGF-dependent actions is unclear. Here, we identified and characterized an *igfbp* gene from sea lamprey, an agnathan, which was separated from the jawed vertebrates 450 million years ago. Phylogenetic and structural analyses suggested that the encoded protein belongs to the IGFBP-3 clade in the IGFBP family. Lamprey IGFBP-3 contains an IGF-binding domain (IBD), nuclear localization signal, and transactivation (TA) domain. Biochemical and functional analyses showed that these domains are all functional. Lamprey IGFBP-3 can bind IGFs and modulate IGF signaling when tested in mammalian cells. Lamprey IGFBP-3 also has the capacity to enter the nucleus and has strong TA activity. Forced expression of lamprey IGFBP-3, but not its IBD mutant, in zebrafish embryos decreased body growth and developmental speed. Lamprey IGFBP-3 inhibited BMP2 signaling in cultured cells and in zebrafish embryos, and this action is independent of its IGF-binding function. These results suggest that lamprey IGFBP-3 has both IGF-dependent and -independent actions and provide new insights into the functional evolution of the IGFBP family.

## Introduction

The insulin-like growth factor (IGF) system is an evolutionarily ancient hormonal pathway that plays fundamental roles in regulating development, growth, reproduction, and aging in vertebrates (1–3). The IGF signaling system consists of IGF ligands, IGF receptors, and IGF-binding proteins (IGFBPs) (2). IGFBPs bind IGF with high affinities and specificity. These proteins have several functions. They act as IGF carrier proteins in the blood and regulate IGF turnover, transport, and distribution. At the target tissues, IGFBPs inhibit and/or potentiate IGF actions by regulating IGF availability to the cell surface of IGF receptor (1–3). IGFBPs also possess IGF-independent actions. Some IGFBPs can be detected in the nucleus and has IGF-independent transactivation (TA) activity (3–7).

When and how these distinct biological functions evolved has long been a mystery. Earlier studies suggested a possible link between the evolution of IGFBP genes and the HOX genes. These genes are neighboring genes on the same chromosomes (8, 9). Daza et al. recently proposed that a single ancestral chordate IGFBP gene underwent a local gene duplication, thereby resulting in a gene pair adjacent to a HOX cluster (10). Subsequently, the IGFBP gene family expanded in the two basal vertebrate tetraploidization events (2R), resulting in the six IGFBP types that are presently found in mammals (10). Indeed, a single IGFBP gene has been found in a basal chordate, amphioxus (8, 10). Amphioxus IGFBP shares major structural characteristics of vertebrate IGFBPs (11). However, the amphioxus IGFBP does not have the capability to bind to modern IGF-I or IGF-II, while it possesses the nuclear localization and transcriptional activation activity (11). Based on these and other data, it has been suggested that the IGF-independent activities may be original and conserved functions, and the IGF-binding functions may have been acquired later in evolution (11). The timing of the emergence of IGF-dependent capacity is not clear.

Sea lamprey (*Petromyzon marinus*), an agnathan, occupies a key position in vertebrate evolution (12). Lamprey and the closely related hagfish are jawless fish, and they diverged from the jawed vertebrates (Gnathostomes) approximately 450 million years ago (12). The phylogenetic position of lampreys makes it an invaluable model for understanding the evolution of genes in vertebrates. Indeed, a single IGF peptide equally related to human IGF-I and IGF-II has been identified in the sea lamprey (13). Likewise, the related Atlantic hagfish also has a single IGF (14). We hypothesized that the IGFBPs may have evolved IGF-dependent actions along with the emergence of IGF in agnathans. To test this hypothesis, we identified and characterized an *igfbp* gene from sea lamprey. This gene encodes lamprey IGFBP-3. Functional analyses showed that lamprey IGFBP-3 has both IGF-dependent and -independent actions.

## Materials and Methods

### Materials

Chemicals and reagents were purchased from Fisher Scientific (Pittsburgh, PA, USA), unless otherwise stated. RNA polymerase, RNase-free DNase, and pGEM-T easy vector were purchased from Promega (Madison, WI, USA). Taq DNA Polymerase, Phusion High-Fidelity DNA Polymerase, and restriction endonucleases were purchased from New England BioLabs (Beverly, MA, USA). M-MLV reverse transcriptase, oligo(dT)_12–18_ primers, and TRIzol^®^ Reagent were purchased from Invitrogen Life Technologies, Inc. (Carlsbad, CA, USA). SMART™ RACE cDNA Amplification Kit was purchased from Clontech Laboratories, Inc. (Mountain View, CA, USA). mMESSAGE mMACHINE kit was purchased from Applied Biosystems/Ambion (Austin, TX, USA). IGF peptides were purchased from GroPep (Adelaide, SA, Australia) and IGFBP-3 and BMP-2 were from R&D systems (Minneapolis, MN, USA). The anti-GFP antibody was purchased from Torrey Pines Biolabs Inc. (East Orange, NJ, USA). Anti phospho-Smad1/5/8 antibody was purchased from Cell Signaling Technology Inc. (Danvers, MA, USA). Total anti-Smad1/5/8 antibody was purchased from Santa Cruz Biotechnology Inc. (Santa Cruz, CA, USA). Digoxigenin and anti-digoxigenin-POD antibodies were purchased from Roche (Indianapolis, IN, USA).

### Experimental Animals

The sea lamprey (*P. marinus*) tissues were provided by Dr. Weiming Li, Michigan State University. Total RNA was extracted from the adult liver and used for cDNA synthesis. Wild-type zebrafish (*Danio rerio*) were maintained on a 14-h light/10-h dark cycle and fed twice a day. Embryos were obtained by natural cross. Fertilized eggs were raised at 28.5°C and staged according to Kimmel et al. (15). 2-Phenylthiourea [0.003% (wt/vol)] was added to the embryo rearing solution in some experiments to inhibit embryo pigment formation. This study was approved by the Institutional Committee on the Use and Care of Animals, University of Michigan. It was carried out in accordance with the recommendation of the Institutional Committee on the Use and Care of Animals, University of Michigan. The protocol was approved by the Institutional Committee on the Use and Care of Animals, University of Michigan.

### Molecular Cloning

The human IGFBP-3 (NM_000598) and zebrafish Igfbp-3 (NM_205751) amino acid (aa) sequences were used as queries to tblastn search the sea lamprey genome database.^1^ The best BLAST hits were collected, and their scaffold locations were determined. The genome sequence surrounding these best reads of short sequence was retrieved and used to predict the sea lamprey IGFBP-3 sequence using GENESCAN.^2^ The predicted protein sequence was searched by blastp.^3^ The full-length sequence of sea lamprey IGFBP-3 cDNA was determined by 5′- and 3′-rapid amplification of cDNA ends (RACE) using the SMART™ RACE cDNA Amplification Kit. The gene specific primers used were 5′GSP1 and 5′NGSP1 for 5′ end amplification, and 3′GSP2 and 3′NGSP2 for 3′ end amplification (Table S1 in Supplementary Material). The genomic structure of lamprey *igfbp-3* was determined by the Blat program^4^ using the cloned full-length cDNA sequence and lamprey Assembly WUGSC 3.0.

### Sequence Alignment and Phylogenetic Analysis

The aa sequence of full-length lamprey IGFBP-3 and that of major IGFBPs (Table S2 in Supplementary Material) was aligned using BioEdit 7.0 (16). The same sequence information was used for subsequent phylogenetic tree construction. The following three bootstrap-supported tree construction methods were used: maximum likelihood (ML), Bayesian posterior probability (BayPP), and neighbor joining (NJ). The ML analysis was performed using ML 3.0 (17). ProtTest 2.4 was used to select the optimal model of aa substitution (18). The robustness of the ML was estimated by 1,000 bootstrap replications using the Jones–Taylor–Thornton (JTT) probability model. BayPP analysis was performed using MrBayes v.3.1 (19), assuming a four-category γ among site rate variation distribution, with uniform priors over trees, branch lengths (0.5), and the ASRV α parameter (0.05–10). A total of 1,000,000 generations were performed with four chains (Markov chain Monte-Carlo) and sampled for every 100 generations. The first 250 samples from each run, a point well past stationarity, were discarded as burn-in. All analyses converged on the same tree and found that the Jones protein model had 100% posterior probability. The NJ tree was constructed using MEGA4 (20) with JTT matrix in NJ method. The reliability of each tree node was assessed by the bootstrap method with 1,000 replications. The constructed trees were then visualized by TreeView (21).

### Synteny Analysis

For synteny analysis, lamprey, zebrafish, and human *IGFBP-3*/*igfbp-3* gene, and their neighboring genes were extracted from Ensembl (Table S3 in Supplementary Material), and a schematic diagram was constructed to show locations of genes on each chromosome or scaffold.

### Plasmid Construction

The lamprey *igfbp-3* open reading frame sequence was amplified by PCR using primers F1 and R1 (Table S1 in Supplementary Material) and cloned into pGEM-T easy vector and sequenced. It was then subcloned into pCS2+ and pCS2 + EGFP vectors using primers F2/R2 and F2/R3 (Table S1 in Supplementary Material), respectively. The lamprey IGFBP-3 IGF-binding domain (IBD) mutant was generated by changing R^86^, P^87^, L^88^, L^91^, and L^92^ to S, A, S, Q, and G using the primers IBDF and IBDR (Table S1 in Supplementary Material), according to Imai et al. (22). The nuclear localization signal (NLS) mutant was generated by mutating ^243^RGRQR^247^ into MDGEA, the corresponding sequence of human IGFBP-1 (23), using primers NLSF and NLSR (Table S1 in Supplementary Material). The transactivation domain (TAD) mutants were generated by changing E^25^/D^28^/E^48^/P^49^/E^70^ into the corresponding residues of human IGFBP-1 (24) by three rounds of mutagenesis using primers TAD1F/TAD1R, TAD2F/TAD2R, and TAD3F/TAD3R (Table S1 in Supplementary Material).

To analyze their possible TA activity, lamprey IGFBP-3 and its N-domain were fused to Gal4 DNA-binding domain (DBD). Briefly, DNA fragments encoding lamprey IGFBP-3 and its N-domain were amplified by PCR using primers shown in Table S1 in Supplementary Material. The PCR products were cloned into the *Bam*HI site of the pBind vector, thereby fusing the fragments in frame to the C-terminus of Gal4DBD.

### Cell Culture, One-Hybrid Transcription Activation Assay, and Subcellular Localization

Human embryonic kidney (HEK) 293T cells, U2 osteosarcoma (U2OS) cells, and HeLa cells, purchased from the American Type Culture Collection (Manassas, VA, USA), were cultured as reported previously (25). HEK293 cells were used in cell proliferation and one-hybrid assays because they are fast growing and easy to transfect. HeLa cells are flattened cells with a large nucleus, and they were used in the subcellular localization experiment. U2OS cells have been previously used to study BMP2 signaling (26).

For one-hybrid transcription activation assay, 500 ng of pBind or pBind-IGFBP DNA and 500 ng of pG5-luc DNA were co-transfected into HEK293T cells as reported previously (24). Twenty-four hours after transfection, cells were washed with PBS and lysed in lysis buffer. The *firefly* and *Renilla* luciferase activities were determined using the Dual-Luciferase Reporter assay system (Promega). For subcellular localization studies, EGFP, lamprey IGFBP-3, and its various mutants–EGFP fusion constructs were transfected into HeLa cells. Transfected cells were fixed by PFA, stained by DAPI, and then imaged as previously reported (26).

### Conditioned Medium (CM), Western Immunoblotting, and Ligand Blotting

To obtain the CM, lamprey IGFBP-3, its various mutants, or human IGFBP-3 plasmid DNA (2 µg) were transfected into HEK 293T cells. Twenty-four hours later, cells were washed three times with PBS and changed to serum-free medium. After incubation for 48 h, the CMs were collected and centrifuged to remove cellular debris. These CMs were subjected to western immunoblotting and ligand blotting analyses as described previously (26). Human IGF-I was labeled with digoxingenin following the published methods (27).

### Cell Proliferation and IGF Signaling Assays

To determine the possible IGF-dependent action of lamprey IGFBP-3, HEK293T cells were plated into 96-well plates in DMEM supplemented with 10% fetal bovine serum. After incubated for 1 day, cells were rinsed three times with serum-free DMEM. One hundred microliters of CMs collected from cells transfected with lamprey IGFBP-3, its IBD mutant, or human IGFBP3 were mixed with 100 µl of DMEM with or without IGF-I (with a final concentration of 100 ng/ml) and incubated for 30 min at room temperature. The mixed media were added to cells. Forty-eight hours later, cell numbers were assayed using a cell counting kit from ZOMANBIO (Beijing) following the manufacturer’s instruction. To study the effect of lamprey IGFBP-3 on IGF-I-induced pAkt signaling, HEK293T cells were plated into 24-well plates and treated as described above. The cells were harvested after 3 h of treatment. The cell lysates were analyzed by immunoblotting using antibodies against pAkt and total Akt.

### BMP2 Signaling Assay

Human U2OS cells were seeded into 24-well plates. After 24 h, the cells were washed and incubated in serum-free medium for 3 h. Then, 100 µl of DMEM containing human BMP2 (20 ng/ml) were mixed with 100 µl of CMs as described and added to the cells. After 3 h, cells were harvested in RIPA buffer containing protease and phosphatase inhibitors (10 µg/ml aprotinin, 10 µg/ml leupeptin, 10 µg/ml pepstatin, 1 mM PMSF, 1 mM sodium orthovanadate, and 1 mM sodium fluoride) and subjected to immunoblotting using antibodies against pSmad1/5/8 and total Smad1/5/8.

### Microinjection Experiment and Real-time Quantitative RT-PCR (qRT-PCR) Analysis

Capped mRNA was synthesized using a commercial mMESSAGE mMACHINE kit and linearized plasmid DNA as template. Two hundred picograms of mRNA encoding EGFP, lamprey IGFBP-3, and its various mutants, 50 pg of mRNA encoding zebrafish Chordin, and 2.5 pg of mRNA encoding zebrafish Bmp2b were microinjected into zebrafish embryos at one to two cell stages, respectively. Intact embryos were used as controls. After microinjection, embryos were raised in embryo rearing medium (28) and kept at 28.5°C. Live embryos were imaged, and body length and somite number were determined at 24 hpf.

For qRT-PCR analysis, total RNA was extracted using TRIzol^®^ Reagent following the manufacturer’s instruction. After DNase treatment, RNA (1 µg) was reverse transcribed with oligo(dT)_18_ primers and M-MLV reverse transcriptase. qRT-PCR was performed in an iCycler iQ Multicolor real-time PCR detection system (Bio-Rad Laboratories, Inc., Hercules, CA, USA) using SYBR^®^ Premix Ex Taq™ (Takara Bio Inc., Dalian, Liaoning). Primer pairs used for qPCR were as follows: tp63F/tp63R for *tp63* and actinF/actinR for β*-actin* (Table S1 in Supplementary Material). Each sample was measured in duplicate. Target gene mRNA levels were calculated using the 2^−ΔΔCT^ method (29) and presented as relative (-fold) values of the control group after being normalized by β*-actin* mRNA levels.

### Statistical Analysis

Values are presented as means ± SD. Differences among groups were analyzed using one-way ANOVA followed by the Turkey’s multiple comparison test. All analyses were performed using GraphPad Prism version 5.01 (San Diego, CA, USA). Significance was accepted at *P* < 0.05 or higher.

## Results

### Identification and Characterization of Sea Lamprey *igfbp-3*

**Figure 1:**
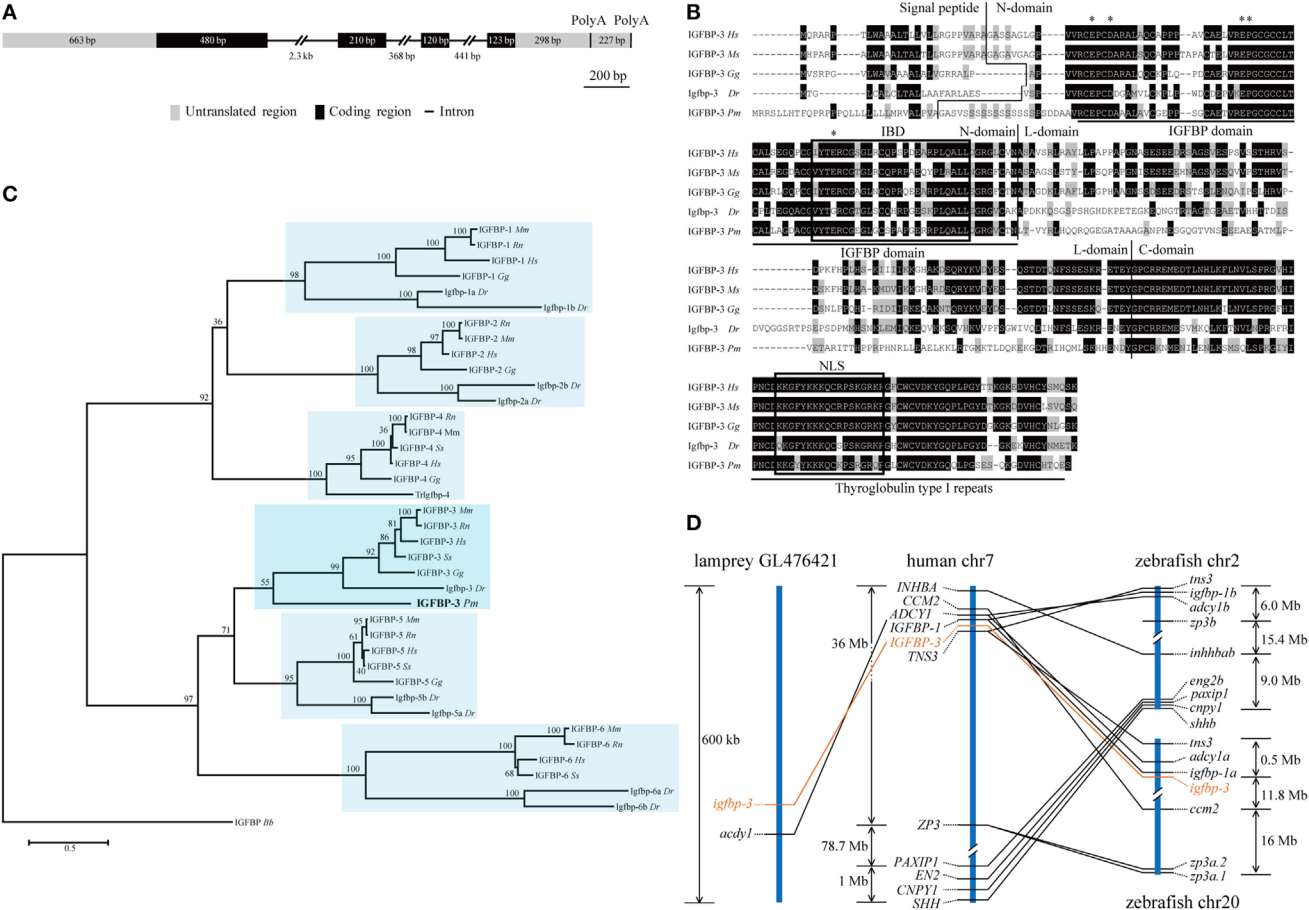
**Structural, phylogenetic, and synteny analysis of lamprey IGFBP-3**. **(A)** The genomic structure of lamprey *igfbp-3*. The two PolyA signals are indicated by vertical lines. **(B)** Comparison of lamprey IGFBP-3 (IGFBP-3 *Pm*) amino acid (aa) sequence with that of human IGFBP-3 (IGFBP-3 *Hs*), mouse IGFBP-3 (IGFBP-3 *Mm*), chicken IGFBP-3 (IGFBP-3 *Gg*), and zebrafish Igfbp-3 (Igfbp-3 *Dr*). Hyphens were inserted for optimal alignment. The identical aas are dark shaded, and similar ones are gray shaded. Signal peptide, N-domain, L-domain, and C-domain are separated by vertical lines. The IGF-binding domain (IBD) and nuclear localization signal (NLS) are indicated by open boxes. The insulin-like growth factor binding protein (IGFBP) and thyroglobulin type I motifs are underlined. The aas known to be critical for transactivation activity in human IGFBPs are indicated by asterisks. **(C)** Phylogenetic analysis. Maximum likelihood (ML) was performed on a matrix composed of full-length aa sequences of major IGFBPs. ML bootstrap values shown are percentage of times that the two clades branched as sisters. Amphioxus IGFBP (IGFBP *Bb*) was used as the outgroup. **(D)** Synteny analysis. The *IGFBP-3/igfbp3* genes are indicated in orange, while their neighboring genes are black. The chromosome or scaffold number and the location of *IGFBP-3/igfbp3* and its neighboring genes are shown. Lines indicate orthologous genes.

Our search of the sea lamprey genome database identified an *igfbp*-like gene (ENSPMAG00000004139) on scaffold GL476421 (previously known as Contig6676.1). Using RACE, the full-length cDNA was obtained and sequenced. The genomic structure was determined. This lamprey gene contains four exons and three introns (Figure 1A). There are two polyadenylation signal sequence (Figure 1A). Thus, the full-length cDNAs are 2,111 bp (KX904869) and 1,894 bp (KX904870), respectively. The putative signal peptide is 32 aa and mature protein is 278 aa with a predicted molecular mass of 29.8 kDa. There are 12 cysteine residues in the highly conserved N-terminal domain (residues 1–101) and 6 cysteine residues in the conserved C-terminal domain (residues 200–278) (Figure 1B). A conserved IGFBP motif is present in its N-domain and a thyroglobulin type 1 repeat in the C-domain (Figure 1B). This protein has the highest sequence identities with IGFBP-3/Igfbp-3 in the IGFBP family (Table S4 in Supplementary Material). Phylogenetic analyses have placed this lamprey IGFBP in the IGFBP-3 clade (Figure 1C; Figures S1 and S2 in Supplementary Material). Therefore, we named this gene as lamprey *igfbp-3*. The lamprey *igfbp-3* is located on scaffold GL476421 (Figure 1D). In human and zebrafish genomes, *IGFBP-3*/*igfbp-3* gene is located next to *IGFBP-1*/*igfbp-1*, arranged with a tail-to-tail orientation (Figure 1D). We were unable to find any *igfbp-1*-like gene in lamprey scaffold GL476421. An *ACDY1* orthologous gene was found in GL476421 in this scaffold (Figure 1D).

### Lamprey IGFBP-3 Has a Functional IBD, NLS, and TAD

**Figure 2:**
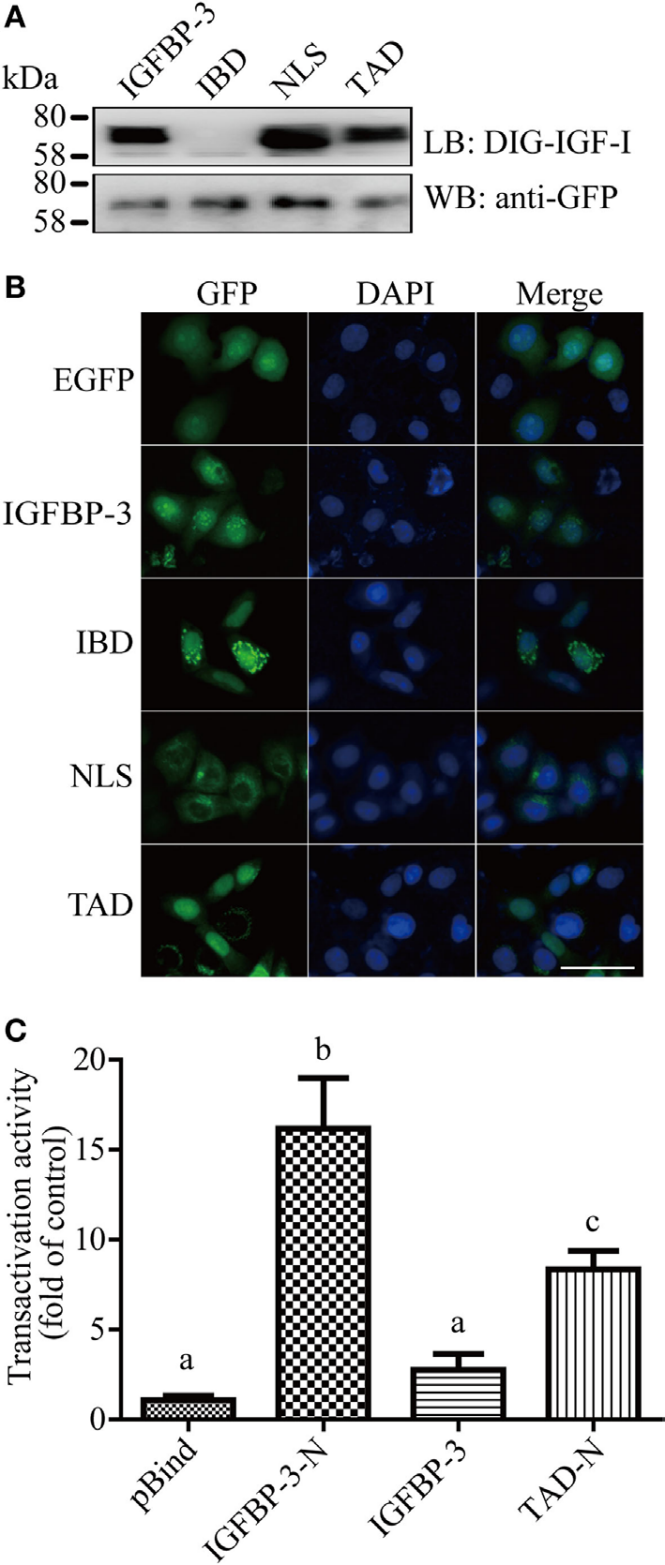
**Lamprey IGFBP-3 contains a functional IBD, nuclear localization signal (NLS), and transactivation domain (TAD)**. **(A)** Ligand blotting (upper panel) and immunoblotting (lower panel) analyses of lamprey IGFBP-3, its IBD, NLS, and TAD mutants. DIG-labeled human IGF-I and anti-GFP antibodies were used, respectively. **(B)** Subcellular localization of lamprey IGFBP-3, its IBD, NLS, and TAD mutants in HeLa cells. The EGFP signal was visualized 24 h after transfection (left panel). Corresponding DAPI staining is shown in the middle column and merged views in the right column. Scale bar = 50 µm. **(C)** Lamprey IGFBP-3 N-domain has strong transactivation (TA) activity. Lamprey IGFBP-3 N-domain (IGFBP-3-N), its TAD mutants (TAD-N), and full-length lamprey IGFPB-3 were fused to pBIND and transfected into HEK293T cells together with a Gal4 reporter plasmid. The TA activity is expressed as fold increase over the pBind control group. Values are expressed as means ± SD (*n* = 3). Groups with different letters are significantly different from each other (*P* < 0.05).

The IGF-binding domain (IBD), TAD, and NLS are found in lamprey IGFBP-3 (Figure 1B). To determine whether these motifs are functional, they were mutated and tested functionally (Figure S3 in Supplementary Material). Ligand blotting assay showed that lamprey IGFBP-3, but not its IBD mutant, was able to bind human IGF-I (Figure 2A). The NLS and TAD mutants were also able to bind IGF-I (Figure 2A). The expression levels of these fusion proteins were similar (Figure 2A). When expressed in HeLa cells, the lamprey IGFBP-3-GFP signal was observed exclusively in the nucleus (Figure 2B). The IGFBP-3 IBD mutant and TAD mutant had similar nuclear. Mutation of NLS, on the other hand, abolished the nuclear localization (Figure 2B), suggesting that the lamprey IGFBP-3 NLS is functional. Next, one-hybrid TA assay was performed. Lamprey IGFBP-3 N-domain (BP3-N) caused a 16-fold increase in GAL4-dependent TA activity (Figure 2C). As the case in human IGFBP-3 (30), the full-length lamprey IGFBP-3 had no such activity, indicating that its activity is masked by its N-domain (Figure 2C). Next, the lamprey TAD sequence was mutated to corresponding sequence in human IGFBP-1, which had no TA activity (24). The TAD mutant had significantly reduced TA activity (Figure 2C). These results indicate that the IBD, NLS, and TAD in lamprey IGFBP-3 are functional.

### Lamprey IGFBP-3 Has IGF-Dependent Action

**Figure 3:**
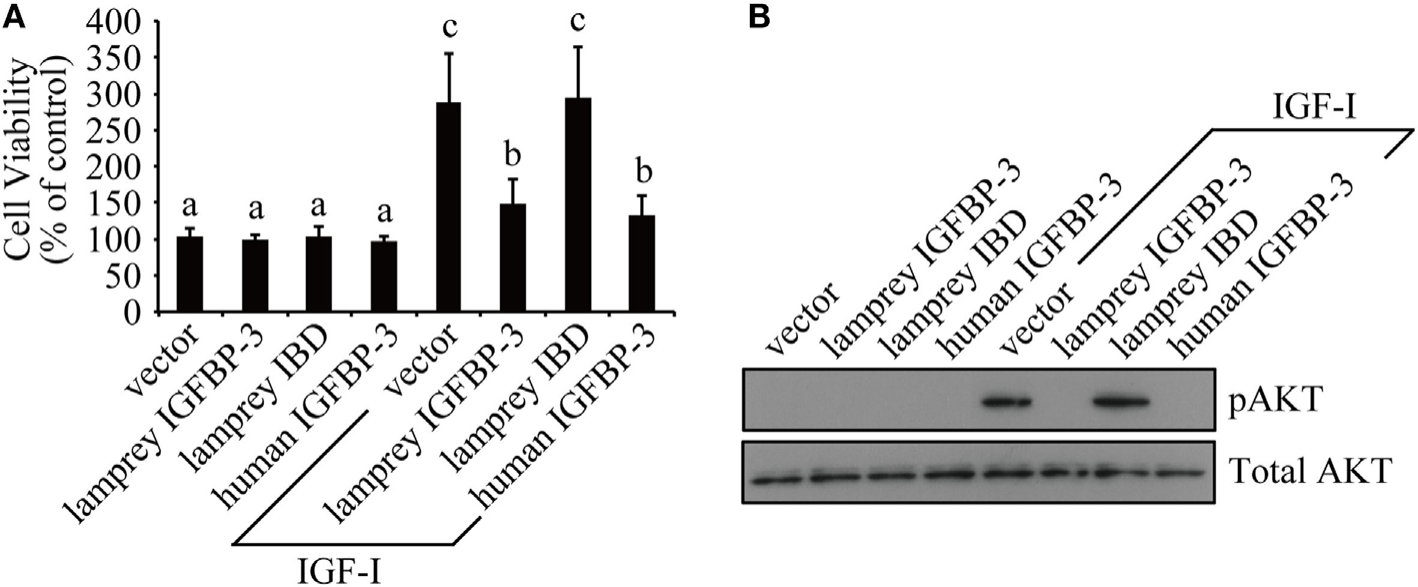
**Lamprey IGFBP-3 has insulin-like growth factor (IGF)-dependent action *in vitro***. **(A)** Lamprey IGFBP-3, but not its IBD mutant, inhibits IGF-I-stimulated cell proliferation. Confluent HEK293T cells were treated with IGF-I together with conditioned media prepared from cells transfected with the empty vector, lamprey IGFBP-3, its IBD mutant, and human IGFBP-3, respectively. The values are expressed as means ± SD (*n* = 3). Groups with different letters are significantly different from each other at *P* < 0.05. **(B)** Lamprey IGFBP-3, but not its IBD mutant, inhibits IGF-I-induced pAkt signaling. HEK293T cells were treated as described in panel **(A)**. Cells were harvested and analyzed by western immunoblotting using indicated antibodies. Similar results were obtained in two additional and independent experiments.

We tested whether lamprey IGFBP-3 has IGF-dependent action in cultured human cells because it is technically difficult to perform functional studies in lamprey. Lamprey IGFBP-3 and its IBD mutant expression plasmids were transfected into HEK293T cells. Human IGFBP-3 plasmid and empty vector transfected cells were used as controls. Addition of IGF-I significantly stimulated HEK293T cell proliferation (Figure 3A). Co-incubation with lamprey IGFBP-3 inhibited IGF-I-induced cell proliferation (Figure 3A). The inhibitory effect was comparable to that of human IGFBP-3 (Figure 3A). Likewise, both lamprey and human IGFBP-3 inhibited IGF-I-induced Akt phosphorylation (Figure 3B). The lamprey IGFBP-3 IBD mutant had no effect on IGF-I-induced cell proliferation or pAkt signaling (Figures 3A,B), suggesting that this action of lamprey IGFBP-3 requires its IBD domain.

**Figure 4:**
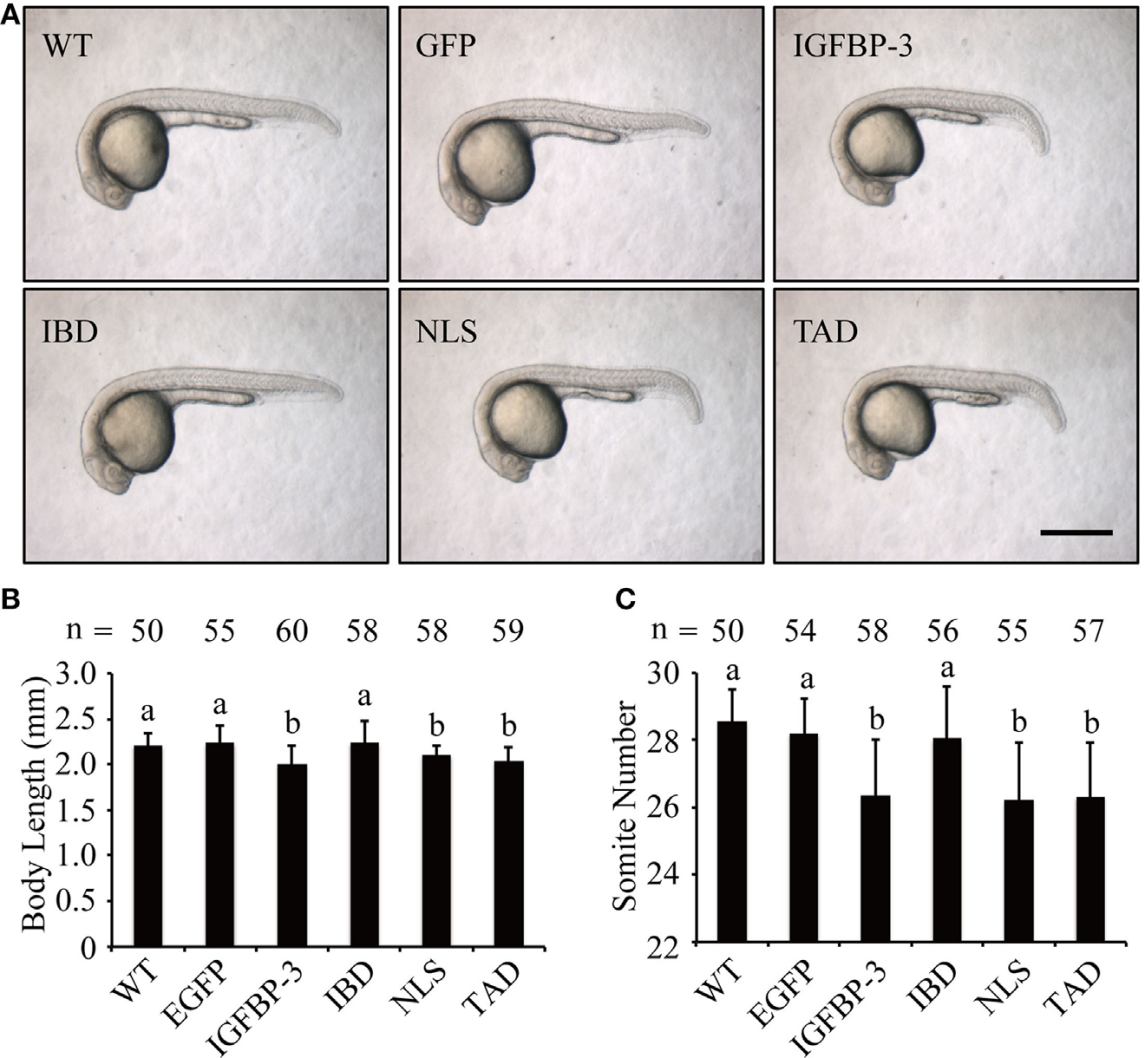
**Lamprey IGFBP-3 has insulin-like growth factor-dependent action *in vivo***. **(A)** Representative images of 24 hpf embryos injected with mRNA encoding the indicated proteins. Wild-type (WT) embryos were used as controls. Scale bar = 0.5 mm. Body length **(B)** and somite number **(C)** of the abovementioned groups were quantified and shown. Values are represented as means ± SD (*n* = 3). The total number of embryos in each group is shown on the top of each column. Groups with different letters are significantly different from each other (*P* < 0.05).

We next investigated whether lamprey IGFBP-3 has IGF-dependent action *in vivo* in zebrafish embryos by mRNA injection experiments. As shown in Figure 4A, the EGFP mRNA-injected embryos were indistinguishable from the WT embryos. Forced expression of lamprey IGFBP-3 significantly decreased body length (Figures 4A,B) and somite number (Figures 4A,C). Similar effects were seen with the TAD and NLS mutants (Figures 4A–C). Mutation of the IBD abolished this action (Figures 4A–C), suggesting that this action is primarily IGF-binding dependent.

### Lamprey IGFBP-3 Has IGF-Independent Function

**Figure 5:**
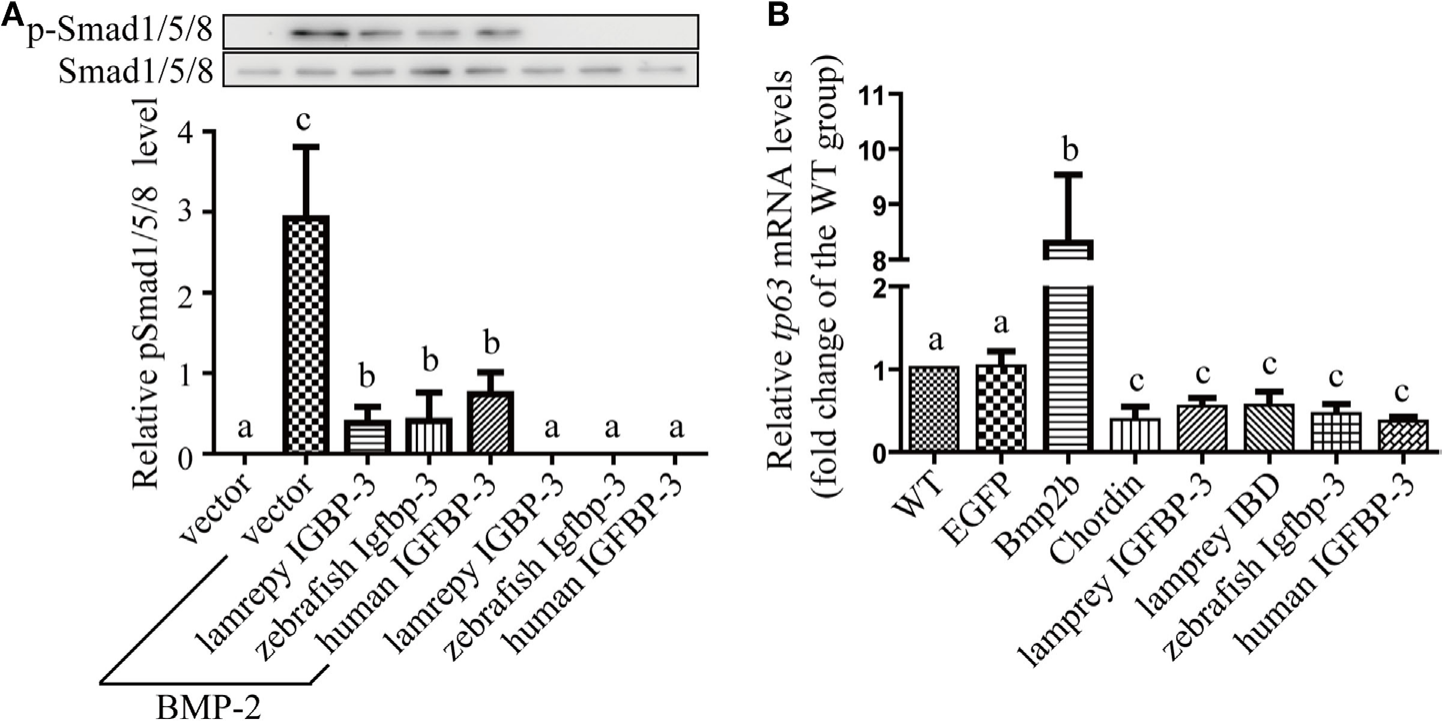
**Lamprey IGFBP-3 antagonizes BMP signaling independently of its insulin-like growth factor-binding function**. **(A)** IGFBP-3 inhibits BMP-2 signaling *in vitro*. Human U2 osteosarcoma cells were treated with 10 ng/ml of human BMP2 plus the indicated conditioned media. Three hours later, the cells were harvested and analyzed using the indicated antibodies. A representative image of western immunoblotting is shown on the top and quantitative results in the lower panel. pSmad1/5/8 level was normalized by total Smad1/5/8 level. Values are expressed as means ± SD (*n* = 3). Groups with different letters are significantly different from each other (*P* < 0.05). **(B)** IGFBP-3 inhibits BMP-2 signaling *in vivo*. Zebrafish embryos were injected with the indicated capped mRNA. Seven hours after injection, embryos were pooled. RNA was extracted and analyzed by quantitative RT-PCR. Wild-type (WT) embryos were used as control. Values are expressed as means ± SD (*n* = 3). Groups with different letters are significantly different from each other at *P* < 0.05.

We have previously reported that human and zebrafish IGFBP-3/Igfbp-3 can antagonize BMP action, and this action is independent of its IGF-binding function (26). To investigate whether lamprey IGFBP-3 has this activity, co-incubation experiments were carried out. Addition of human BMP-2 strongly induced Smad1/5/8 phosphorylation in cultured human U2OS cells (Figure 5A). Co-incubation with CMs containing lamprey IGFBP-3, zebrafish Igfbp-3, or human IGFBP-3 strongly inhibited BMP-2-induced Smad1/5/8 phosphorylation (Figure 5A). The CMs from the empty vector transfected cells had no such effect (Figure 5A).

Next, mRNA injection experiments were carried out. Injection of Bmp2b mRNA resulted in an eightfold and highly significant increase in the mRNA levels of *tp63* (Figure 5B), a known Bmp target gene (31). Injection of Bmp2b with Chordin mRNA, a well-known Bmp antagonist, significantly reduced *tp63* mRNA expression (Figure 5B). Likewise, injection lamprey IGFBP-3 mRNA resulted in a significant decrease in *tp63* mRNA expression (Figure 5B). This effect was comparable to those of human IGFBP-3 and zebrafish Igfbp-3 (Figure 5B). Importantly, the lamprey IGFBP-3 IBD mutant had similar effect (Figure 5B). These results suggest that lamprey IGFBP-3 has the ability to antagonize Bmp signaling and this action is IGF independent.

## Discussion

Insulin-like growth factor-binding proteins were initially discovered based on their ability to directly bind with IGF-I and/or -II. All vertebrate IGFBPs studied to date can bind to IGF-I and/or IGF-II with high affinity (2). Subsequent studies revealed that several IGFBPs, including IGFBP-3, also possess IGF-binding-independent functions. Although all known IGFBPs are secreted proteins, some are also found in the nucleus and possess IGF-independent TA activities (24, 30, 32, 33). A recent study suggested that while amphioxus IGFBP has the nuclear localization and TA activity, it lacks the IGF-binding function (11). This has led to the idea that the nuclear localization and transcription activation activity of IGFBPs appeared early and the IGF-binding function may have been acquired by opportunistic gain-of-functional mutations later in evolution (11). When did the IGF-dependent action emergence, however, was unclear. In this study, we sought to determine whether IGF-dependent action was evolved before or after divergence of agnathans from jawed vertebrates by identifying and characterizing IGFBP-like molecules from sea lamprey. In this study, we identified and cloned an *igfbp* gene from this species. Phylogenetic and structural analyses showed that this lamprey gene is most closely related to mammalian IGFBP-3.

IGFBP-3 is a multifunctional protein. It is the predominant IGFBP in the blood and functions as the major IGF carrier protein in the circulation. IGFBP3 is also expressed in many peripheral tissues and has also been shown to inhibit and/or potentiate the actions of IGF in a wide variety of tissues (32). Moreover, IGFBP-3 has been reported to possess IGF-independent actions. Mammalian and other vertebrate IGFBP-3 studied to date all contain several functional motifs/domains, including IBD, NLS, and TAD. Structural analysis suggested that all these motifs/domains are conserved in lamprey IGFBP-3. The primary IGF-binding site (R/KPLXXLL) is located in the N-domain in mammalian and zebrafish IGFBP-3s (22, 26). This sequence is perfectly conserved in lamprey IGFBP-3. Our ligand blot analysis results showed that lamprey IGFBP-3 indeed can directly bind to human IGF-I. Mutation of the IBD sequence in lamprey IGFBP-3 impaired its IGF-binding action. Importantly, lamprey IGFBP-3 has the ability to modulate IGF action when tested in zebrafish embryos *in vivo* and human cells *in vitro*, while its IBD mutant cannot. These structural and functional analysis results argue strongly that the IGF-binding function of IGFBPs have evolved prior to the emergence of agnathans. This speculation is in good agreement with the current understanding of the evolution history of insulin/IGF peptides. It is believed that the IGFs are derived from an ancestral insulin-like gene that has duplicated and diverged many times throughout metazoan evolution, yielding insulin and the IGFs, in addition to a number of insulin-like peptides (34). To date, IGF-like molecules have not been identified in any invertebrate. Agnathans such as lamprey and hagfish apparently have only one IGF that has features characteristic of both IGF-I and IGF-II (13, 14). In primitive jawed vertebrates such as elasmobranch (e.g., shark), two distinctive IGF-like peptides appear: one is more similar to mammalian IGF-I molecules, while the other is more like IGF-II (35). These observations have lead to the proposal that IGF-like molecule emerged prior to or at agnathans and then separated into IGF-I and IGF-II in jawed fish. Our finding on the IGF-binding and IGF-dependent action of lamprey IGFBP-3 agrees well with this scenario. It should be pointed out that human IGF-I was used, and the biological assays were performed in human cells and zebrafish embryos. Future studies are needed to further address this issue using pure and biologically active lamprey IGF and in lamprey.

Mammalian and zebrafish IGFBP-3/Igfbp-3 has a functional NLS and can be found in the nucleus of cultured tumor cells (3, 4, 26). When tested in mammalian cells, human and zebrafish IGFBP-3 both showed strong TA activity (26, 30). These functions have been attributed to a conserved NLS in the C-domain and a TAD in the N-domain of IGFBP-3. In this study, we investigated whether the NLS and TAD are present and functional in lamprey IGFBP-3. Our results suggested that lamprey IGFBP-3 N-domain has TA activity when tested in mammalian cells. A 50% reduction in TA activity was observed when several conserved acidic residues were mutated, suggesting that other residues may also contribute to the TA activity. Lamprey IGFBP-3 can localize into the nuclei of mammalian cells, and this nuclear localization activity was abolished when NLS was mutated. In comparison, the IBD mutant and TAD mutant protein showed normal nuclear localization activity. These results suggest that the TAD and NLS are conserved and functional in agnathans.

We have previously reported that human and zebrafish IGFBP-3/Igfbp-3 can antagonize BMP action in zebrafish embryos, and this action is independent of its IGF-binding function (26). We used this *in vivo* assay system to further investigate the ligand-independent action of lamprey IGFBP-3. Forced expression of lamprey IGFBP-3 increased the mRNA expression of *tp63*, a known Bmp target gene. This action was indeed independent from IGF-binding because lamprey IGFBP-3 IBD mutant had similar effect. When tested in cultured human cells, lamprey IGFBP-3 had a similar inhibitory effect on BMP2-induced Smad signaling. This effect was comparable to those of human IGFBP-3 and zebrafish Igfbp-3. Collectively, these data indicate that lamprey IGFBP-3 has an evolutionarily conserved ability to antagonize Bmp signaling and this action is IGF independent.

In summary, lamprey IGFBP-3 can bind IGFs and modulate IGF signaling when tested in mammalian cells. Forced expression of lamprey IGFBP-3, but not its IBD mutant, in zebrafish embryos decreased body growth and developmental timing. Lamprey IGFBP-3 also has the capacity to enter the nucleus and has strong TA activity. When tested in human cells and zebrafish embryos, lamprey IGFBP-3 inhibited BMP2 signaling, and this action is independent of its IGF-binding function. These findings suggest that lamprey IGFBP-3 has both IGF-dependent and -independent actions and have provided new insights into the functional evolution of the IGFBP family. Future studies will determine the presence, structure, and function of other IGFBPs in lamprey to gain a better understanding of the evolution of the IGFBP gene family.

## Author Contributions

YZ conducted all the experiments and analyzed the data. CD and YZ designed the experiments and wrote the manuscript.

## Conflict of Interest Statement

The authors declare that the research was conducted in the absence of any commercial or financial relationships that could be construed as a potential conflict of interest.

## Footnotes

^1^http://www.ensembl.org/Petromyzon_marinus/Info/Index.

^2^http://genes.mit.edu/GENSCAN.html.

^3^http://blast.ncbi.nlm.nih.gov/Blast.cgi.

^4^http://genome.ucsc.edu/cgi-bin/hgBlat.
