# Supplementary material for: Lamprey IGF-Binding Protein-3 Has IGF-Dependent and -Independent Actions
Source: Front Endocrinol (Lausanne). 2017 Jan 18;7:174. doi: 10.3389/fendo.2016.00174 (PMC5241279; doi:10.3389/fendo.2016.00174)
Supplement: Supplementary file 1 [file Data_Sheet_1.DOC]

Supplementary Material

**Lamprey IGF Binding Protein-3 Has IGF-dependent and -independent Actions**

**Yingbin Zhong^1,2^ and Cunming Duan^2*^**

^1^School of Biology & Basic Medical Sciences, Medical College, Soochow University, Suzhou, Jiangsu, China;

^2^Department of Molecular, Cellular, and Developmental Biology, University of Michigan, Ann Arbor, MI, USA;

**^*^Correspondence**:

Cunming Duan

Department of Molecular, Cellular, and Developmental Biology, University of Michigan

Ann Arbor, MI 48109, USA

Fax: 734-647-0884

E-mail: [cduan@umich.edu](mailto:cduan@umich.edu)

Supplementary Table 1. Primers used

| Primer | Sequence |
| --- | --- |
| 5’GSP1 | GTTCTCCATGTTTTTGCGGCACGGTCC |
| 3’GSP2 | GGCGGGTGCTAATCCCAACGA |
| 5’NGSP1 | GTCCCCCTTCTCCTTCTGGTCCAAGGTC |
| 3’NGSP2 | CGGGCAGGGCACTGTGAACTCG |
| F1 | GGCGTGCGTTAAGCCTCACTAT |
| R1 | TCACCTTGTCGGCTTAAGTC |
| F2 | CGGGATCCGCCACCATGCGCCGCAGTCTCCTCCACACG |
| R2 | CGGAATTCCTAATCACTCTCCTGAGTATGACAATG |
| R3 | CGGAATTCGATCACTCTCCTGAGTATGACAATG |
| IBDF | CGGGGAACGGTCGGCCTCCCAGGCGCAGGGCGACGGCCGC |
| IBDR | GCGGCCGTCGCCCTGCGCCTGGGAGGCCGACCGTTCCCCG |
| NLSF | GTGCAAGCCATCGATGGACGGGGAAGCTGGCCTCTGCTGG |
| NLSR | CCAGCAGAGGCCAGCTTCCCCGTCCATCGATGGCTTGCAC |
| TADF1 | GCGGCCGTGCGATGCGCGCCGTGCTCCGCAGCAGCGCTGGCC |
| TADR1 | GGCCAGCGCTGCTGCGGAGCACGGCGCGCATCGCACGGCCGC |
| TADF2 | GCGGCGTCTACACGGCGCGCTGCGGCGAGGGG |
| TADR2 | CCCCTCGCCGCAGCGCGCCGTGTAGACGCCGC |
| TADF3 | GCCGAGACGGTGCGGTCGGCCGGCTGCGGCTGCTGC |
| TADR3 | GCAGCAGCCGCAGCCGGCCGACCGCACCGTCTCGGC |
| pBindFL | CGCGGATCCGTGCCAGCGTCTCCTCCTCGTC |
| pBindNR | CGCGGATCCCTAGCAGACGCCGCGGCCGTCGA |
| pBindR | CGCGGATCCCTACTAATCACTCTCCTGAGTATG |
| tp63F | AGAGATGGTCAGGTTTTGGG |
| tp63R | GTGCTGTTTGCGGATGC |
| actinF | AGAGTCCATCACGATACCAG |
| actinR | AGAGTCCATCACGATACCAG |

Supplementary Table 2. Genes used for phylogenetic analysis and sequence alignment.

| Species | Protein | GENE ID |
| --- | --- | --- |
| Zebrafish (*Danio rerio*) | DrIgfbp-1a | [NM_173283](http://www.ncbi.nlm.nih.gov/nuccore/NM_173283.3) |
|  | DrIgfbp-1b | 窗体顶端  NM_001098257 窗体底端 |
|  | DrIgfbp-2a | 窗体顶端  NM_131458窗体底端 |
|  | DrIgfbp-2b | 窗体顶端  NM_001126464 窗体底端 |
|  | DrIgfbp-3 | 窗体顶端  NM_205751窗体底端 |
|  | DrIgfbp-5a | 窗体顶端  NM_001098754窗体底端 |
|  | DrIgfbp-5b | 窗体顶端  NM_001098754窗体底端 |
|  | DrIgfbp-6a | 窗体顶端  NM_001161401窗体底端 |
|  | DrIgfbp-6b | NM_001161402窗体底端 |
| Human (*Homo sapiens*) | HsIGFBP-1 | NM_000596 |
|  | HsIGFBP-2 | NM_000597 |
|  | HsIGFBP-3 | NM_000598 |
|  | HsIGFBP-4 | NM_001552 |
|  | HsIGFBP-5 | NM_000599 |
|  | HsIGFBP-6 | NM_002178 |
| Mouse (*Mus musculus*) | MmIGFBP-1 | NM_008341 |
|  | MmIGFBP-2 | NM_008342 |
|  | MmIGFBP-3 | NM_008343 |
|  | MmIGFBP-4 | NM_010517 |
|  | MmIGFBP-5 | NM_010518 |
|  | MmIGFBP-6 | NM_008344 |
| Rat (*Rattus norvegicus*) | RnIGFBP-1 | NM_013144 |
|  | RnIGFBP-2 | NM_013122 |
|  | RnIGFBP-3 | NM_012588 |
|  | RnIGFBP-4 | NM_001004274 |
|  | RnIGFBP-5 | NM_012817 |
|  | RnIGFBP-6 | NM_013104 |
| Bovine (*Bos taurus*) | BbIGFBP-1 | NM_174554 |
|  | BbIGFBP-2 | NM_174555 |
|  | BbIGFBP-3 | NM_174556 |
|  | BbIGFBP-4 | NM_174557 |
|  | BbIGFBP-5 | NM_001105327 |
|  | BbIGFBP-6 | NM_001040495 |
| Chicken (*Gallus gallus*) | GaIGFBP-1 | NM_001001294 |
|  | GaIGFBP-2 | NM_205359 |
|  | GaIGFBP-3 | NM_001101034 |
|  | GaIGFBP-4 | NM_204353 |
|  | GaIGFBP-5^#^ | XM_422069 |
| Pig (*Sus scrofa*) | SsIGFBP-3 | NM_001005156 |
|  | SsIGFBP-4 | NM_001123129 |
|  | SsIGFBP-5 | NM_214099 |
|  | SsIGFBP-6 | NM_001100190 |
| Xenopus (*Xenopus tropicalis*) | XtIGFBP-1 | NM_001033946 |
|  | XtIGFBP-2 | NM_001100237 |
|  | XtIGFBP-4^*^ | ENSXETG00000021361 |
|  | XtIGFBP-5 | NM_001016042 |
| Rainbow trout (*Oncorhynchus mykiss*) | OmIGFBP-1 | NM_001124561 |
|  | OmIGFBP-2a | NM_001124649 |
|  | OmIGFBP-2b | NM_001124557 |
|  | OmIGFBP-4 | DQ146967 |
|  | OmIGFBP-5 | NM_001124652 |
|  | OmIGFBP-6 | NM_001124560 |
| Chimpanzee (*Pan troglodytes*) | PtIGFBP-3^#^ | XM_519084 |
| Sheep (*Ovis aries*) | OaIGFBP-3 | NM_001159276 |
| Stickleback (*Gasterosteus aculeatus*) | GaIGFBP-3a^*^ | ENSGACT00000006412 |
|  | GaIGFBP-3b^*^ | ENSGACG00000013090 |
| Tetraodon (*Tetraodon nigroviridis*) | TnIGFBP-3a^*^ | ENSTNIT00000009389 |
|  | TnIGFBP-3b^*^ | ENSTNIT00000005941 |
| Fugu (*Takifugu rubripes*) | TrIGFBP-3^*^ | ENSTRUT00000033398 |
| Medaka (*Oryzias latipes*) | OlIGFBP-3^*^ | ENSORLT00000021455 |
| Sea lamprey (*Petromyzon marinus*) | PmIGFBP-3 | ENSPMAG00000004139 |
| Spotted gar (*Lepisosteus oculatus*) |  |  |
| Amphioxus (*Brnachiostoma belcheri*) | BbIGFBP | FJ971406 |

#, GenBank predicted sequence; *, Ensembl predicted sequence.

Supplementary Table 3. Genes used in the synteny analysis

| Species | Gene | GENE ID^*^ |
| --- | --- | --- |
| Sea lamprey (*Petromyzon marinus*) | *igfbp-3* | ENSPMAG00000004139 |
|  | *adcy1* | ENSPMAG00000004160 |
| Human (*Homo sapiens*) | *INHBA* | ENSG00000122641 |
|  | *CCM2* | ENSG00000136280 |
|  | *ADCY1* | ENSG00000164742 |
|  | *IGFBP-1* | ENSG00000146678 |
|  | *IGFBP-3* | ENSG00000146674 |
|  | *TNS3* | ENSG00000136205 |
|  | *ZP3* | ENSG00000188372 |
|  | *PAXIP1* | ENSG00000157212 |
|  | *EN2* | ENSG00000164778 |
|  | *CNPY1* | ENSG00000146910 |
|  | *SHH* | ENSG00000164690 |
| Zebrafish (*Danio rerio*) | *tns3* | ENSDARG00000087773 |
|  |  | ENSDARG00000095654 |
|  |  | ENSDARG00000031911 |
|  | *igfbp-1a* | ENSDARG00000014947 |
|  | *igfbp-1b* | ENSDARG00000038666 |
|  | *igfbp-3* | ENSDARG00000014859 |
|  | *adcy1a* | ENSDARG00000068890 |
|  | *adcy1b* | ENSDARG00000088634 |
|  | *ccm2* | ENSDARG00000013705 |
|  | *zp3a.1* | ENSDARG00000042129 |
|  | *zp3a.2* | ENSDARG00000042130 |
|  | *zp3b* | ENSDARG00000039828 |
|  | *paxip1* | ENSDARG00000005606 |
|  | *eng2b* | ENSDARG00000038868 |
|  | *cnpy1* | ENSDARG00000003757 |
|  | *shhb* | ENSDARG00000038867 |

^*^, Gene ID in Ensembl.

Supplementary Table 4. Amino acid sequence identities between sea lamprey IGFBP-3 and other vertebrate IGFBPs/Igfbps

| **Species**  **Protein** | **Human** | | **Mouse** | | **Rat** | **Bovine** | **Chicken** | **Xenopus** | **Trout** | **Zebrafish** |
| --- | --- | --- | --- | --- | --- | --- | --- | --- | --- | --- |
| **IGFBP-1**  **/Igfbp-1** | 26 | 29 | | 29 | | 26 | 32 | 30 | 26 | 32/26^b^ |
| **IGFBP-2**  **/Igfbp-2** | 30 | 31 | | 30 | | 28 | 28 | 28 | 26/26^a^ | 24/25^c^ |
| **IGFBP-3**  **/Igfbp-3** | 45 | 43 | | 43 | | 43 | 42 | - | - | 39 |
| **IGFBP-4**  **/Igfbp-4** | 32 | 30 | | 30 | | 32 | 28 | 28^*^ | 16^†^ | - |
| **IGFBP-5/**  **Igfbp-5** | 38 | 37 | | 39 | | 37 | 20^#^ | 35 | 35 | 34/35^d^ |
| **IGFBP-6**  **/Igfbp-6** | 26 | 25 | | 26 | | 27 | - | - | 26 | 25/15^e^ |

#, Comparison was made using GenBank predicted sequences;

*, Comparison was made using ensemble predicted sequences;

†, Comparison was made using corresponding partial sequences;

a, Amino acid sequence identities between sea lamprey Igfbp-3 and rainbow trout Igfbp-2a/2b;

b, Amino acid sequence identities between sea lamprey Igfbp-3 and zebrafish Igfbp-1a/1b;

c, Amino acid sequence identities between sea lamprey Igfbp-3 and zebrafish Igfbp-2a/2b;

d, Amino acid sequence identities between sea lamprey Igfbp-3 and zebrafish Igfbp-5a/5b;

e, Amino acid sequence identities between sea lamprey Igfbp-3 and zebrafish Igfbp-6a/6b.


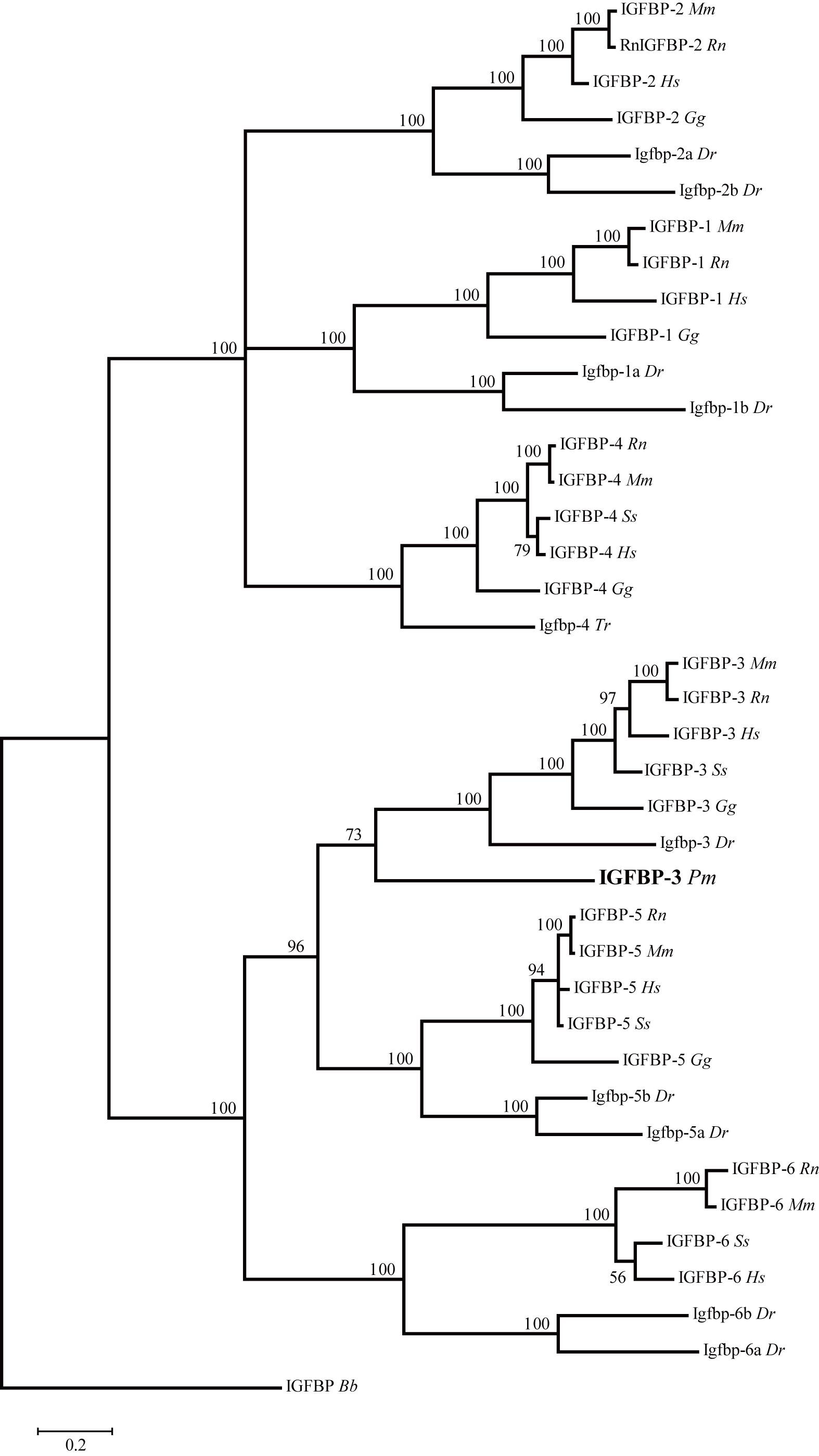


**Supplementary Figure 1**. Phyologentic analysis. Bayesian inference analysis was performed by MrBayes v.3.1. A total of 1,000,000 Bayesian generations were performed using the Jones-Taylor-Thornton (JTT) probability model. Bayesian posterior probability (BayPP) values shown in each branch are percentage of times that the two clades grouped as sisters. Amphioxus IGFBP was used as the outgroup. The results indicate that the newly identified sea lamprey IGFBP-3 is most closely related to the IGFBP-3 subgroup.


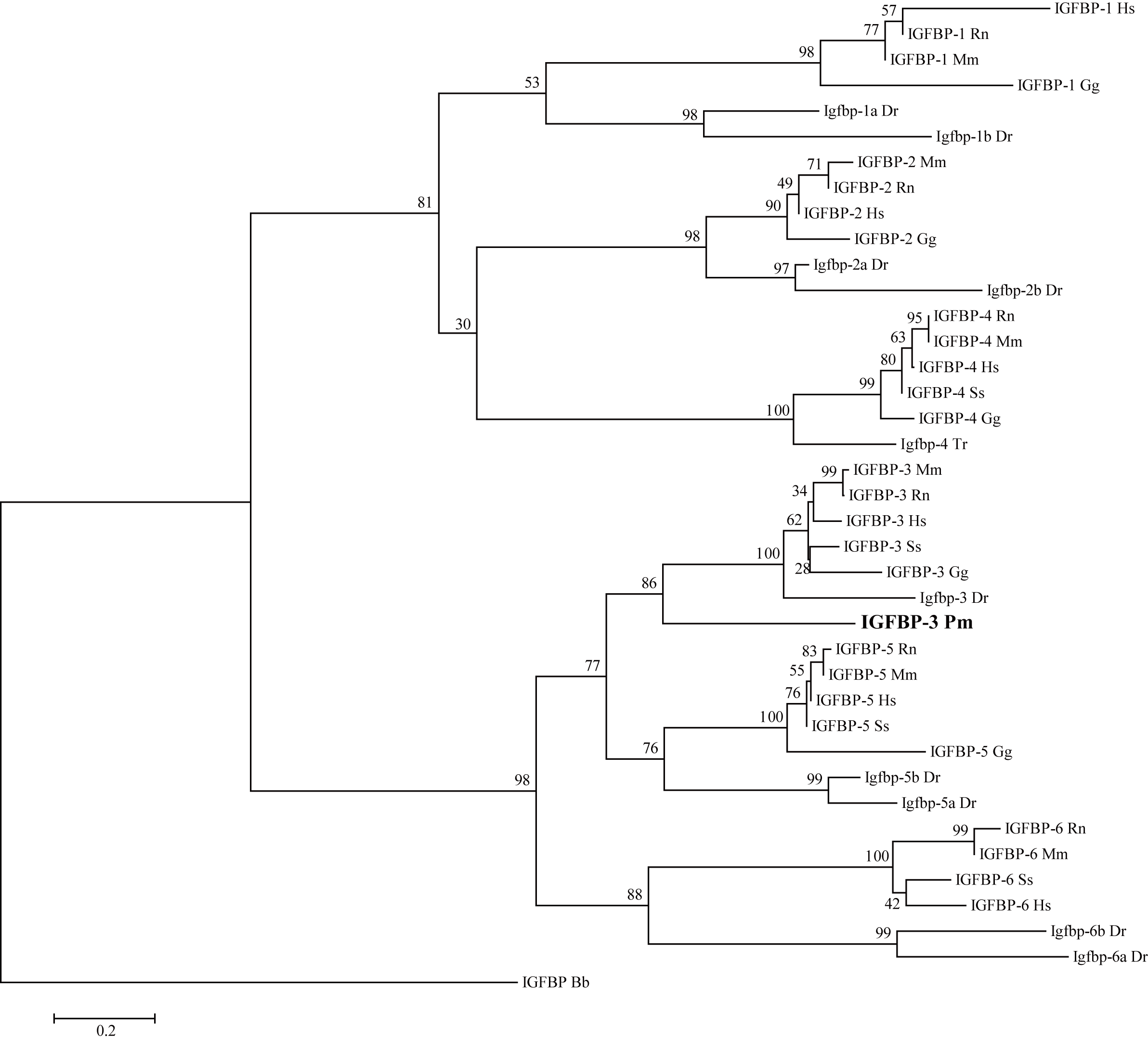


**Supplementary Figure 2.** Phyologentic analysis by Neighbor-joining method. Full-length sequences of IGFBPs were analyzed by MEGA4 with JTT matrix in neighbor-joining method. The reliability of each node was assessed by the bootstrap method with 1000 replications. Numbers on branches are percentage of times that the two clades branched as sisters. Amphioxus IGFBP gene was used as the outgroup. The results indicate that the newly identified IGFBP is most closely related to the IGFBP-3 subgroup.


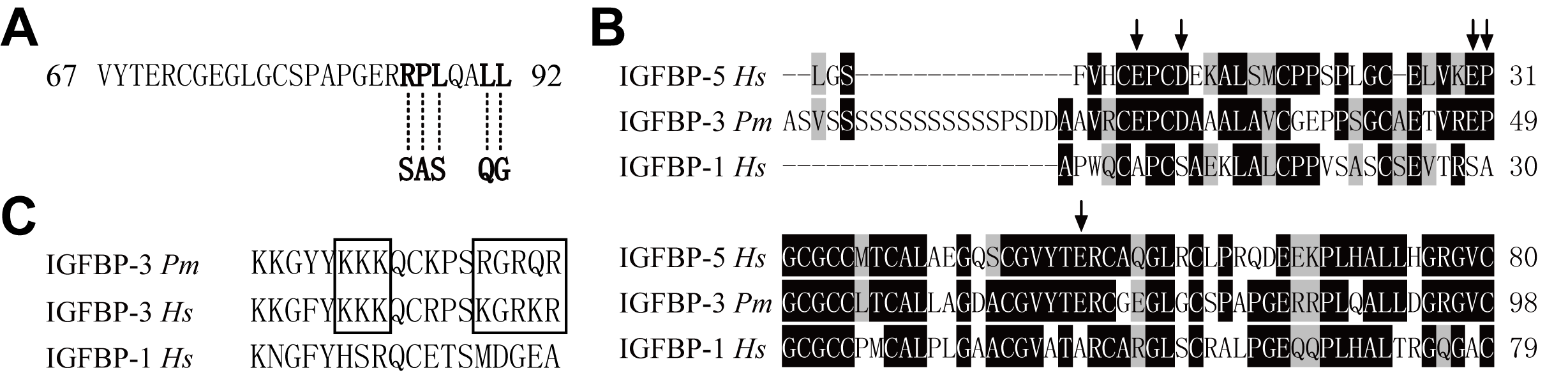


**Supplementary Figure 3**. Design of lamprey IGFBP-3 IBD, TAD, and NLS mutants. **(A)** The IBD mutant was generated by replacing the critical amino acids (in bold) in the lamprey IGFBP-3 IGF binding domain into the indicate amino acids. **(B)** Alignment of lamprey IGFBP-3 (IGFBP-3 *Pm*), human IGFBP-5 (IGFBP-5 *Hs*), and IGFBP-1 (IGFBP-1 *Hs*) N-domain sequence. The TAD mutant was generated by changing E^25^, D^28^, E48, P49, and E^70^ into corresponding human IGFBP-1 residues. **(C)** Alignment of lamprey IGFBP-3 (IGFBP-3 *Pm*), human IGFBP-3 (IGFBP-3 *Hs*), and human IGFBP-1 (IGFBP-1 *Hs*) partial C-domain sequence. The basic amino acids in the NLS were boxed. RGRQR in lamprey IGFBP-3 was changed into the corresponding sequence (MDGEA) in human IGFBP-1.
