# Supplementary figures and images for: Lamprey IGF-Binding Protein-3 Has IGF-Dependent and -Independent Actions
Source: Front Endocrinol (Lausanne). 2017 Jan 18;7:174. doi: 10.3389/fendo.2016.00174 (PMC5241279; doi:10.3389/fendo.2016.00174)

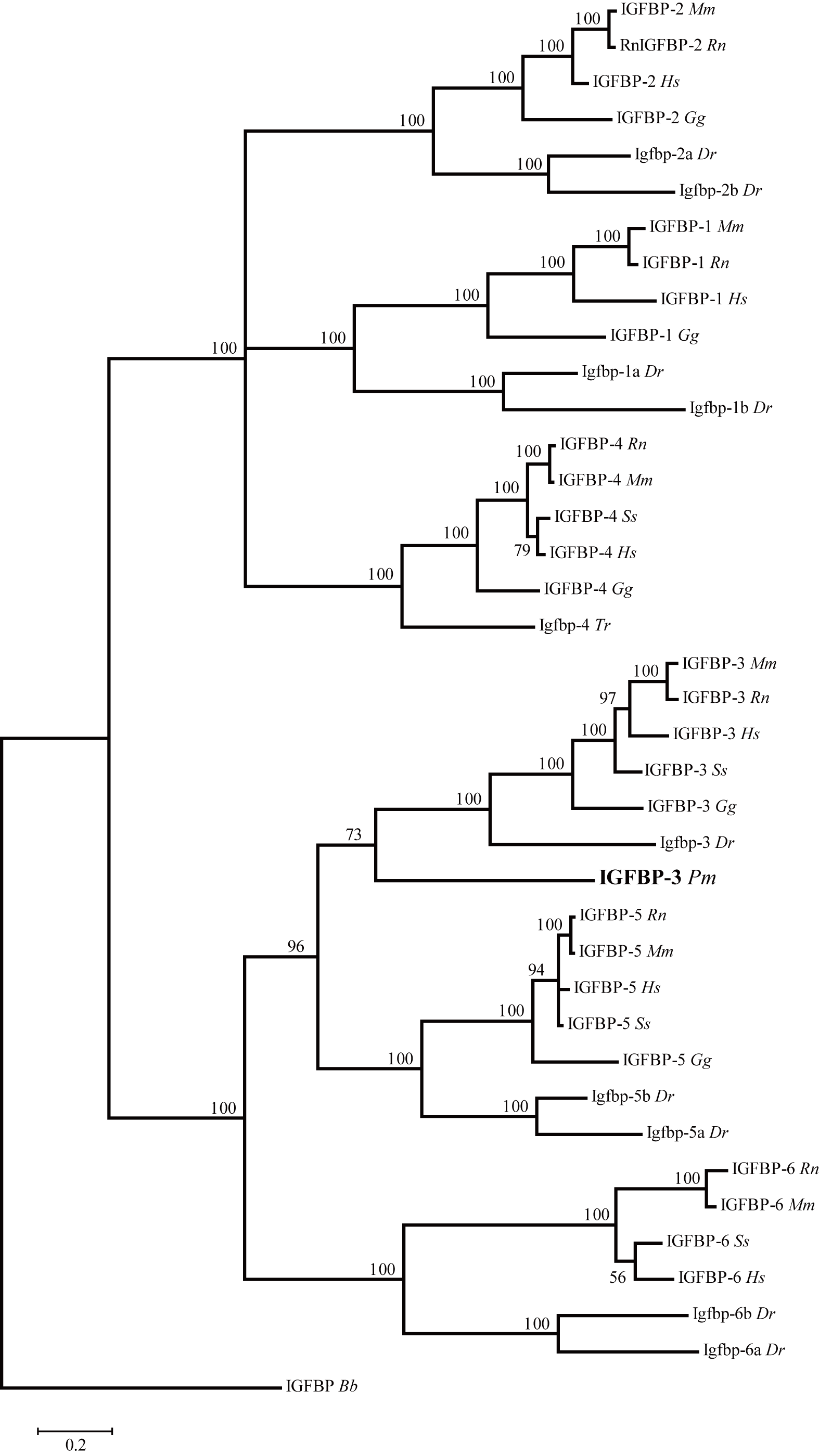

Supplement: Supplementary file 2 [file Image_1.TIF]

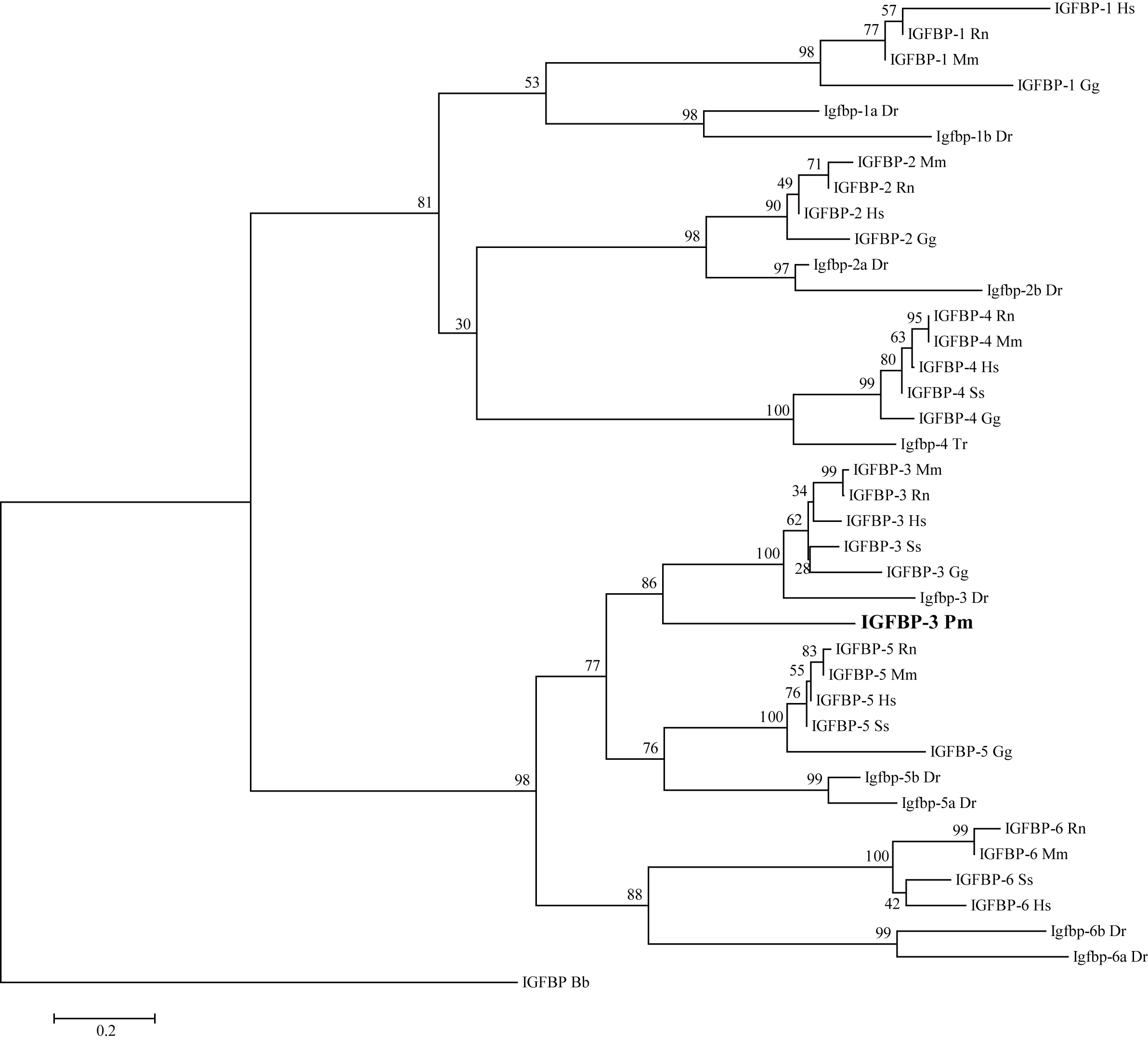

Supplement: Supplementary file 3 [file Image_2.TIF]

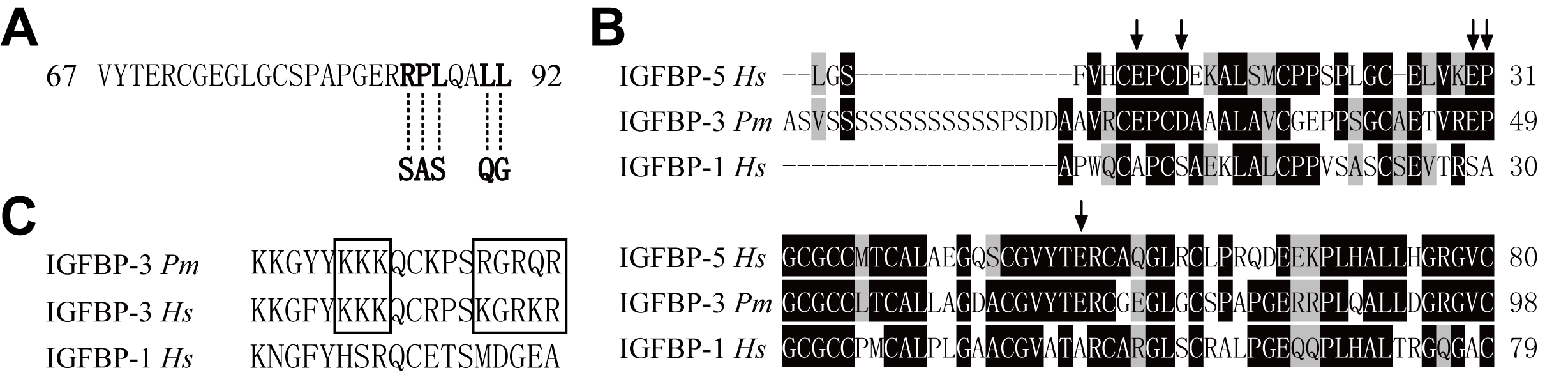

Supplement: Supplementary file 4 [file Image_3.TIF]
